# Complete Genome Sequence of Klebsiella pneumoniae Siphophage Skenny

**DOI:** 10.1128/MRA.01036-19

**Published:** 2019-09-26

**Authors:** Jacob Gramer, Sarah Kenny, Heather Newkirk, Mei Liu, Jason J. Gill, Jolene Ramsey

**Affiliations:** a Center for Phage Technology, Texas A&M University, College Station, Texas, USA; DOE Joint Genome Institute

## Abstract

Klebsiella pneumoniae is a Gram-negative opportunistic pathogen and a leading cause of antibiotic-resistant nosocomial infections. The genome sequence of siphophage Skenny, which infects K. pneumoniae, is described here. Skenny encodes 78 genes and is closely related to *Klebsiella* phages KPN N141 and MezzoGao, which are T1-like phages.

## ANNOUNCEMENT

Klebsiella pneumoniae is a Gram-negative member of the family Enterobacteriaceae and is a leading cause of nosocomial infections (1). K. pneumoniae is becoming increasingly resistant to the most common antibiotic treatments (2), and phage therapy provides a promising alternative treatment (3). Here, we describe the genome sequence of the T1-like siphophage Skenny.

Bacteriophage Skenny was isolated from filtered (0.2 μm) activated sludge collected at a wastewater treatment plant in College Station, TX, due to its ability to form plaques on lawns of the pKpQIL plasmid-cured derivative of K. pneumoniae strain 1776c (4). The host was grown aerobically in tryptic soy broth or agar (Difco) at 37°C, and phage propagation was done using the soft-agar overlay method (5). Skenny genomic DNA was purified by the shotgun library preparation protocol modification of a Promega Wizard DNA clean-up system, prepared with a TruSeq Nano low-throughput kit, and sequenced using Illumina MiSeq 250-bp paired-end reads with v2 500-cycle chemistry (6). The 453,880 total reads in the phage-containing index were quality controlled with FastQC (http://www.bioinformatics.babraham.ac.uk/projects/fastqc/), trimmed by the FastX toolkit v0.0.14 (http://hannonlab.cshl.edu/fastx_toolkit/), and assembled into a single contig at 530-fold coverage using SPAdes v3.5.0 with default parameters (7). The contig was confirmed to be complete by PCR (forward primer, 5′-GTTGCTCGGAACCTGGATAA-3′; reverse primer, 5′-CCCGGTAGAAATGCCAGATAA-3′) and Sanger sequencing. Genes were predicted with GLIMMER v3.0 and MetaGeneAnnotator v1.0 in the Web Apollo instance hosted by the Center for Phage Technology (8–10). We searched for potential tRNAs with ARAGORN v2.36 (11). Gene functions were predicted using InterProScan v5.22-61 and TMHMM v2.0, as well as BLAST with a 0.001 maximum expectation value cutoff versus the NCBI nonredundant and UniProtKB Swiss-Prot/TrEMBL databases (12–15). Annotation tools, which were used at default parameters, are found in the Galaxy instance at https://cpt.tamu.edu/galaxy-pub/ (16). Independent protein analysis was performed using HHPred from the HHSuite v3.0, with the HHblits ummiclust30_2018_08 database for multiple sequence alignment generation and the PDB_mmCIF70 database for modeling (17). Rho-independent termination sites were searched with TransTermHP v2.09 (18). The morphology of Skenny was determined using transmission electron microscopy at the Texas A&M Microscopy and Imaging Center by staining with 2% (wt/vol) uranyl acetate (19).

Skenny is a siphophage with a 49,935-bp genome. The genome coding density is 90.7% and the G+C content is 50.8%, which is significantly lower than the G+C content of 57.2% for the host K. pneumoniae (4). Using PhageTerm, Skenny is predicted to undergo pac-type headful packaging (20). By using progressiveMauve 2.4.0, we found that Skenny shares high nucleotide identity with various T1-like phages, including 96% and 98% identity with K. pneumoniae phages KPN N141 (GenBank accession number MF415412) and MezzoGao (GenBank accession number MF612072), respectively (21). There are 78 genes predicted to encode proteins for Skenny, but no tRNAs were detected. Many genes with predicted function in Skenny also shared BLASTp similarity to phage T1. Within the tail assembly chaperones for the tape measure protein, there is a frameshift sequence analogous to the G and GT proteins of phage λ (22).

### Data availability.

The genome sequence and associated data for phage Skenny were deposited under GenBank accession number MK931444, BioProject accession number PRJNA222858, SRA accession number SRR8869225, and BioSample accession number SAMN11360417.
